# Teratogenic Evaluation of 80% Ethanol Extract of *Embelia schimperi* Vatke Fruits on Rat Embryo and Fetuses

**DOI:** 10.1155/2022/4310521

**Published:** 2022-10-22

**Authors:** Zelalem Animaw, Kaleab Asres, Selamawit Tadesse, Hirut Basha, Samson Taye, Abiy Abebe, Eyob Debebe, Girma Seyoum

**Affiliations:** ^1^Department of Anatomy, College of Health Sciences, Addis Ababa University, Addis Ababa, Ethiopia; ^2^Department of Pharmaceutical Chemistry and Pharmacognosy, College of Health Sciences, Addis Ababa University, Addis Ababa, Ethiopia; ^3^Department of Pathology, St Paul's Millennium Medical College, Addis Ababa, Ethiopia; ^4^Traditional and Modern Drug Research Directorate, Ethiopian Public Health Institute, Addis Ababa, Ethiopia

## Abstract

**Introduction:**

*Embelia schimperi* Vatke (family *Myrsinaceae*) is a commonly consumed anthelminthic plant in Ethiopia. The plant has significant efficacy in treating intestinal worms. However, there are limited data about the safety/toxicity of the plant. Moreover, the teratogenic effect of the plant is not yet well studied despite significant number of Ethiopian mothers consuming herbal medication during their pregnancy.

**Purpose:**

This study aimed to evaluate the teratogenic effect of the hydroalcoholic extract of *E. schimperi* fruit on rat embryos and fetuses.

**Methods:**

Pregnant albino Wistar rats were treated with 80% hydroalcoholic fruit extract of *E. schimperi* at 250 mg/kg, 500 mg/kg, and 1000 mg/kg dosage, whilst the controls were pair-fed and ad libitum groups. Maternal food intake, maternal weight gain, number of implantations, number of prior resorptions, fetal viability, fetal weight, fetal and embryonic crown-ramp length, placental weight, placental gross morphology and histopathology of placental tissue, number of somites, embryonic system, gross/visceral morphological malformations, and ossification centers were evaluated as teratogenicity indices.

**Results:**

The crude extract of *E. schimperi* did not exhibit a significant difference in most developmental indices including the development of a circulatory system, nervous system, and musculoskeletal systems among treated animals and the controls. However, histopathological evaluation of placentas from the treatment groups showed that inflammatory reactions and calcifications compared to the pair-fed and ad libitum controls.

**Conclusion:**

Administration of the 80% hydroalcoholic extract of *E. schimperi* fruit during the period of organogenesis in rats did not show a significant toxic effect on embryonic and fetal developmental indices. However, it might affect the structural integrity of the placenta as it is evidenced by inflammatory reactions and calcifications of decidua basalis of rat placenta.

## 1. Introduction

According to the World Health Organization (WHO) and Center for Disease Control and Prevention (CDC), helminthic infestations are part of the neglected tropical diseases (NTDs) commonly affecting people living in low socioeconomic status [1]. Poor hygiene status and low coverage of deworming are common risk factors that put populations at risk of helminthic infestation [2]. The problem has been affecting different vulnerable groups like immunocompromised individuals and school children in Ethiopia [2, 3]. More importantly, pregnant women are at high risk of acquiring helminthic infestation [4]. This in turn predisposes them for pregnancy complications that can affect the fetomaternal outcomes [5]. Despite such clinical and public health concern, a significant number of individuals are highly dependent on herbal medications to treat helminthic infestation due to shortage of modern health service access and conventional medication [6]. Among these herbs, *Embelia schimperi* Vatke, family *Myrsinaceae*, is the commonly consumed anthelminthic plant [7]. Supporting the traditional claim, scientific investigations have proved that the fruit extract of *E. schimperi* possessed genuine activity against intestinal worms [8, 9]. However, there are limited data available about the safety/toxicity of the plant [10]. Moreover, the toxic effect of the plant extract on the developmental process is not yet well studied even though Ethiopian pregnant mothers, who are highly vulnerable to helminthic infestation, consume herbal medicine at least once during their pregnancy [11].

## 2. Materials and Methods

### 2.1. Plant Collection and Authentication

Fresh fruits of *E. schimperi* were collected from Debre Markos localities located 305 km northwest of the capital Addis Ababa, Ethiopia. The plant was identified and authenticated by a botanist at the Department of Plant Biology and Biodiversity Management, Addis Ababa University, where a voucher specimen (collection number ZA001) was deposited for future reference.

### 2.2. Preparation of the Crude Extract

Fruits of *E. schimperi* were dried at room temperature for two weeks at the herbarium of Traditional and Modern Medicine Research Directorate (TMMRD) of Ethiopian Public Health Institute (EPHI). The dried fruits were then ground using an electronic grinding mill. The powder was first defatted using *n*-hexane in a Soxhlet apparatus. This was followed by maceration of 100 g of the marc 1 L of 80% ethanol in a ratio of 1 : 10 (w/v). As a result, a yield of 12.8% dried crude extract was obtained.

### 2.3. Experimental Animal Preparation

Nulliparous female albino Wistar rats weighing 220–250 g, which were not subjected to the previous experimental procedures, were used. The animals were maintained in a stainless-steel metallic cage at room temperature (22 ± 3°C) with a relative humidity of 50%–60% under a controlled alternating 12-hour light-dark cycle. Animals were acclimatized for 5 days prior to the experiment. During the period of adaptation, all the animals received food (pellet) and tap water ad libitum. Factors thought to bring fetal losses which were not treatment-related such as unnecessary handling of pregnant animals and stress from external factors like noise was minimized [12].

### 2.4. Mating of Experimental Animals

Mating was carried out by introducing a male albino Wistar rat of proven fertility into a cage containing two virgin female rats. Day-1 of gestation was determined the next morning after microscopic examination of vaginal smear to look for the presence of sperm cells. For those female rats with no sperm cells, a vaginal smear was repeated and examined the next morning. The male rat was maintained inside the same cage until confirmation of pregnancy [13, 14].

### 2.5. Dose Preparation and Administration

Pregnant rats were grouped randomly into five groups containing 10 pregnant rats in each group. The first three groups were treated with 250 mg/kg (Group I), 500 mg/kg (Group II), and 1000 mg/kg (Group III) of the crude extract suspended in distilled water, (1 ml/100 gm of body weight) [12] with free access to tap water and food. The animals in Group IV were categorized as a pair-fed control group and were supplied with the mean daily food intake of the previous three groups of animals with distilled water. The pair-fed control group was intended to evaluate if there would be a difference in the outcome variables due to food intake variation in the previous three groups. Group V animals were labeled as ad libitum control group taking food and water without restriction. The ad libitum group was designed to evaluate the effect of animal handling during administration of the crude extract. The doses were calculated based on previous studies [7, 10]. The extracts were administered through oral gavage. Experiments were carried out on 12 days old rat embryos and 20 days old fetuses. During both experiments, the treatment period was from day-6 through day-12 of gestation. The rationale behind selecting this period for treatment was due to the fact that this period represents a period of active embryogenesis and organogenesis. As a result, it was designated as the critical periods of rat development.

### 2.6. Cage Side Evaluation

Cage side clinical observation of animals was done once daily for possible signs of behavioral and physical changes throughout the experimental period. Coma, convulsions and tremors, eyes, feces consistency, fur and skin, mortality, mucous membrane, respiration, salivation, somatomotor activity, behavior pattern, and urination (colour) were the parameters during cage side evaluation as signs of toxicity. Cage side evaluation was done every 30 minutes for the first 4 hours after administration of the crude extract [15].

In order to avoid bias generally, we used double blinding manner during handling of lab animals, administration of the crude extract, and evaluation of the outcome variables. Each of the aforementioned activities were carried out anonymously by different individuals who had no clue about the grouping of animals and the given treatments.

### 2.7. Day-12 Experiment

The purpose of this experiment was to evaluate the teratogenic effect of *E. schimperi* on 12 days old rat embryo. At the end of the treatment period (day-12 of gestation), the dams were anesthetized by injecting sodium pentobarbital 150 mg/kg intraperitoneally [16, 17]. Laparotomy was done to reveal the gravid uterine horns which was later dissected along the antimesometrial border to divulge the developing embryos. With the aid of GXM-XTL3T101 dissecting stereomicroscope and fine forceps, the membranes were removed along with the adjacent maternal tissue to reveal the embryo surrounded by a yolk sac. At this juncture, the yolk sac circulation became clearly visible and was evaluated thoroughly. The yolk sac was then removed to evaluate embryonic developmental indices like the embryonic nervous system, sensory organs, and musculoskeletal systems. These variables were examined according to the Brown and Fabro morphological scoring system [18] (Supplemental file) which was adopted for *in vivo* teratogenicity studies by Belete et al. [19] and Abebe et al. [16].

### 2.8. Day-20 Experiment

These experiments were carried out in 20 days old rat fetuses. The goal was to evaluate the potential toxicity of *E. schimperi* on fetomaternal outcomes and fetal developmental indices in near-term rat fetus. The weight of each pregnant animal was recorded on 1st, 6th, 12th, and 20th day of gestation. Food intake for every 24 hours was weighed the next morning at a constant time starting from day-1 of gestation up to day of sacrifice. Similarly, administration of the plant extract was done daily at a constant time [20].

**Figure 1:**
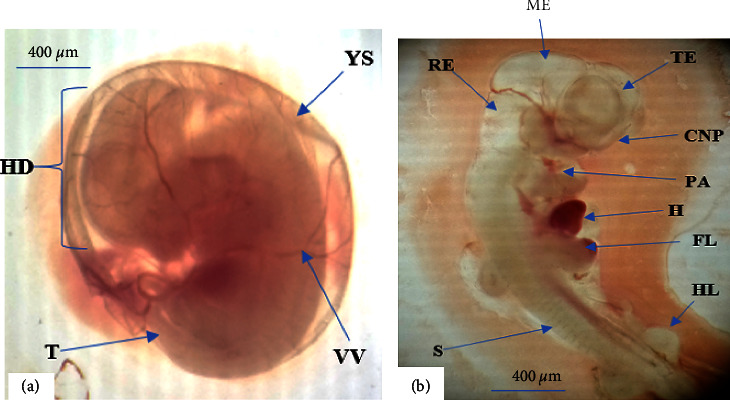
12 days old embryos from rats treated with 1000 mg/kg crude extract of *E. schimperi*. (a): Embryo enclosed by its yolk sac (YS) with visible vitelline vessels (VV), distinguishable head (HD), and tail (T) regions. (b): CNP (cranial neuropores/closed), FL (fore limb), HL (hind limb), ME (mesencephalon), PA (pharyngeal apparatus), RE (rhombencephalon), S (somite), and TE (telencephalon).

On the day of sacrifice (day-20 of gestation), the dams were anesthetized by injecting sodium pentobarbital. Laparotomy was done to reveal the gravid uterus (Figure 1). Gravid uteruses were explanted immediately after the euthanasia and placed in a broad Petri dish. A careful incision was made along the antimesometrial border of the uterus guided by a dissecting microscope (GXM-XTL3T101 stereo microscope). The fetuses were revealed by removing the fetal membranes and detaching them from their respective placentas. After revealing the gravid uterus, the number of implantation sites and prior resorptions was counted and recorded. Alive/dead fetuses were counted after applying gentle pressure on them. Once the fetal membranes and other maternal tissues were removed, the fetuses were weighed using a calibrated digital balance (Mettler AE160). Crown-ramp length (CRL) was measured for every fetus. Placental weight was also recorded before histopathological tissue processing.

### 2.9. Placenta Gross Morphology and Histopathology

Three placenta sample tissues were taken from each animal for gross examination and further histopathological analysis. Samples were initially fixed with 10% formaldehyde for 24 h. Then, tissue processing was carried out using an automatic tissue processor (Leica, TP 1020, Germany). The steps were arranged to start with dehydration by ascending gradient of alcohol concentration followed by clearing and impregnation by xylene and melted paraffin wax, respectively. The tissues were then embedded in paraffin wax and ready for sectioning. The thickness of the tissue to be sectioned by the microtome was adjusted to 5 *μ*m for light microscopy. Finally, the tissues were stained by a hematoxylin and eosin technique [16, 21]. Later, the histological slides were examined by a senior pathologist under a light microscope for indices of functional as well as structural changes in the placenta [22, 23].

### 2.10. External Gross Morphological Evaluation

Fetuses were revealed by removing the fetal membranes and detaching them from their respective placentas. Afterward, each fetus was fully examined for the presence of gross structural malformations of craniofacial development, limb development, vertebral column, tail development, and external genitalia.

### 2.11. Soft Tissue Evaluation

After fetuses were fixed in Bouin's solution (picric acid 75%, formalin 25%, and glacial acetic acid 5%), visceral/soft tissue evaluation was conducted by a free-hand razor blade sectioning technique based on a recommendation by Seegmiller et al. as a modified Willison's technique [16]. The legs and tail were removed at the place of their attachment to the trunk before making a transverse cut between the jaws by a sharp blade. This will help to evaluate the palate for any cleft. Subsequently, coronal slices were made through the head to evaluate the presence of hydrocephalus, ventricular enlargement of the brain, and nasal septum defect [16]. Further transverse sections were made along the trunk to evaluate the possible existence of cardiovascular, respiratory, and abdominal defects.

### 2.12. Skeletal Staining and Evaluation

This experiment was designed to study the effect of *E. schimperi* on the process of bone formation on 20 days old rat fetus after staining the bones of the rat fetus. Three fetus/litter were sampled and further processed based on the Rigueur and Lyons method [24]. The initial step was euthanizing the fetus with pentobarbital followed by tissue permeabilization and skin removal facilitated by bathing for 30 sec in 60°C hot water. Afterwards, evisceration was done by making an abdominal incision. The eviscerated samples were immersed in a solution containing a fixative solution, 90% ethanol, for 24 h. The samples were then transferred to a container filled with 1% potassium hydroxide (KOH) for a purpose of soft tissue removal. Subsequently, the samples were stained for 24 h by a solution of alizarin red (0.005%) at 4°C to obtain an optimum level of skeletal staining. For those samples presumed to be over stained by alizarin red, Mall's solution (79% distilled water, 20% glycerin, and 1% KOH) was used as a correction chemical. Finally, each sample was stored in an increasing concentration gradient of glycerol till examination. Hyoid bone, sternum, ribs, vertebrae, and bones, the upper and lower limbs were assessed against Nash and Persaud's skeletal scoring chart [25].

### 2.13. Statistical Analysis

Data were entered and analyzed using the Statistical Package for Social Science (SPSS) software version 24. The statistical results were exhibited in terms of the mean (*μ*) and the standard deviation (SD). One-way analysis of variance (ANOVA) with the post Hoc (Turkey) test and Chi-square test at *P* < 0.05 level of significance was employed to look over significant statistical differences among experimental groups. Results of placental histopathology were presented qualitatively based on predefined parameters [19, 26].

### 2.14. Ethical Consideration

Ethical approval letter (Ref no. AAUMF03-008) was obtained from the Institutional Review Board (IRB) of the College of Health Sciences, Addis Ababa University with a protocol number 021/19/Anat in biddableness with OECD test guideline (TG-414/2018) [12]. Experimental animals were humanly handled based on the guidelines for ethical conduct in the care and use of nonhuman animals in research by American Psychological Association (APA) [27]. TMMRD/EPHI laboratory standards were also strictly followed in humanely disposing sacrificed rats.

## 3. Results

### 3.1. Cage-Side Clinical Observation

Daily cage-side clinical observation was done and recorded carefully. However, there were no significant behavioral and physical signs of toxicity observed for the whole duration of treatment period. Moreover, there was neither abortion nor maternal death report.

### 3.2. Day-12 Experiment

#### 3.2.1. Embryonic Outcomes

As illustrated in Table 1, the embryonic developmental outcomes revealed that there is no statistically significant difference in morphological score, number of somites, and CRL parameters within the treatment and control groups.

#### 3.2.2. Embryonic Development Indices

*(1) Circulatory system*. Yolk sac circulation and heart development were evaluated as embryonic development indices of the circulatory system. The result showed that there is no statistically significant difference concerning the aforementioned indices between the experimental groups treated with *E. schimperi* and their control counterparts (Table 2) (Figure 1).

*(2) Nervous system and sense organs*. As indicated in Table 3 and Figure 1, the nervous system and sense organs development were assessed by examining the following indices: caudal neural tube, hindbrain, forebrain, auditory system, and optic system. However, the observation found that there was no significantly associated retarded development among the experimental and control groups in the nervous system and sense organs development.

*(3) Musculoskeletal system*. As depicted in Table 4, musculoskeletal development indices of the experimental rats revealed that none of the parameters showed statistically significant retardation among the experimental groups compared to the control groups.

### 3.3. Day-20 Experiment

#### 3.3.1. Food Intake and Weight Gain

The food intake of pregnant rats was measured daily for each group starting from day 1 up to the day of sacrifice. Nevertheless, the overall weight gain was computed by subtracting the initial weight (day-0) from the weight measurement on the day of sacrifice (day-20). As illustrated in Table 5, there is no statistically significant difference in both food consumption and weight gain between the groups during the period of administration and even till the day of scarification.

#### 3.3.2. Pregnancy Outcomes

**Figure 2:**
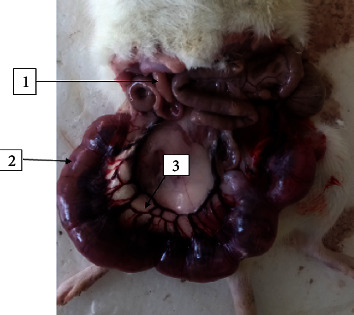
A photograph of rat gravid uterus (1: intestine, 2: gravid uterus, and 3: uterine vessels).

After exposing the uterine horns, the gravid uterus was assessed for pregnancy outcome variables (Figure 2). As shown in Table 6, the number of implantation sites was counted and turns not to have a statistically significant difference between the treatment and control groups. There was also no significant difference in the number of resorption sites and live fetuses among the experimental groups. Each implantation site held an alive fetus.

*(1) Fetal outcomes*. Fetal weight, placental weight, and crown-ramp length were measured as parameters of fetal outcomes. However, none of them possessed a significant statistical difference among the five groups (Table 7).

**Figure 3:**
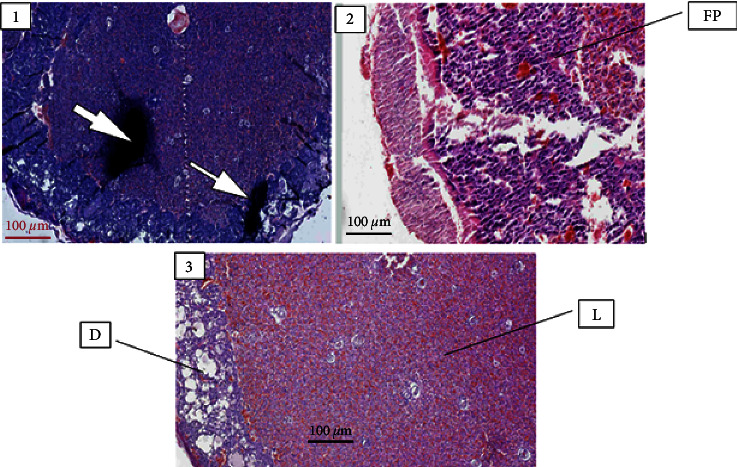
Photo micrograph depicting the following: (1) a calcified placental tissue (arrow) from rats treated with 1000 mg/kg of *Embelia schimperi*, (2) fibroprulent tissue (FP) from rats treated with 500 mg/kg, and (3) normal histology of placenta from the control rats; decidual layer (D), labyrinthine zone (L).

*(2) Gross morphology and histopathology of placenta*. As seen in Figure 3, light microscopic examination of placental histopathology revealed that tissues from Group I and Group II experimental groups exhibited inflammation (focal fibro-purulent exudate and hemorrhage) on the decidual layer. Moreover, animals treated with 1000 mg/kg crude extract of *E. schimperi* showed placental tissue calcification in addition to fibropurulent exudate and hemorrhage. Placenta samples taken from the two control groups did not show any pertinent finding deviating from the normal histology. As illustrated in Table 8, quantitative analysis of histopathological parameters showed a statistically significant difference in the occurrence of inflammatory reactions in placental tissues from *E. schimperi*-treated rats when compared to the control groups. Additionally, calcification is also significantly observed in placentas from rats treated with 1000 mg/kg of *E. schimperi* crude extract.

#### 3.3.3. External and Visceral Morphology

**Figure 4:**
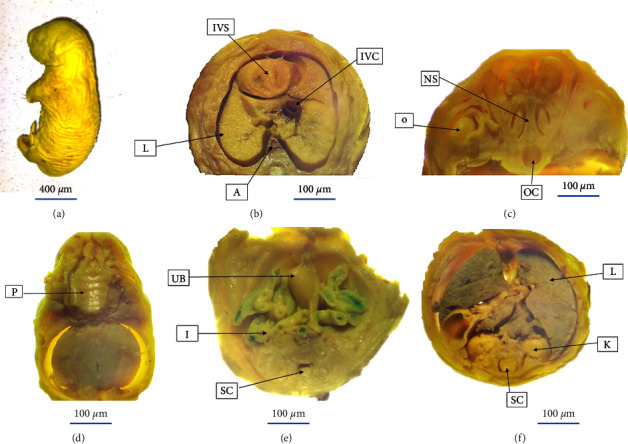
Visceral structures before and after sectioning at different level of the body based on free-hand razor blade sectioning technique. (a) A matured fetus before sectioning. (b) (A: aorta, IVC: inferior vena cava, IVS: interventricular septum, and L: lung). (c) (NS: nasal septum, (O) orbit, and OC: oral cavity). (d) (P: palate). (e) (I: intestine, SC: spinal cord, and UB: urinary bladder). (f) (K: kidney, (L) lung, and SC: spinal cord).

Each fetus was examined carefully for the presence of external structural/morphological malformations after explanting it at the gestational age of 20 days. However, there was no significant treatment-related external morphological defect observed across the experimental groups and control groups (Table 9). Soft tissue/visceral evaluation of fetuses fixed with Bouin's solution revealed that there were no visible abnormalities of visceral structures among fetuses born from rats treated with *E. schimperi* and controls (Figure 4).

#### 3.3.4. Skeletal Evaluation

**Figure 5:**
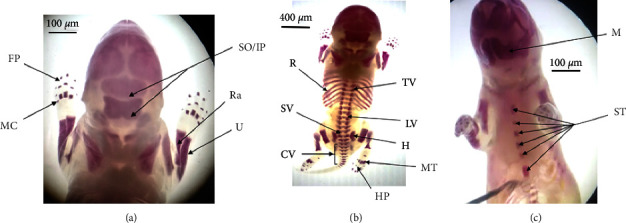
Ossification centers of 20 days old rat fetuses stained with alizarin red. (a) (FP; Forelimb phalanges, IP: interparietal, MC: metacarpus, Ra: radius, So: supraoccipital, and U: ulna). (b) (CV: caudal vertebrae, H: hip bone, HP: hindlimb phalanges, LV: lumbar vertebrae, MT: metatarsus, R: ribs, SV: sacral vertebrae, and TV: thoracic vertebrae). (c) (M: mandible and ST: sternum).

As shown in Tables 10 and 11 and Figure 5, the evaluation of skeletal ossification on rat fetuses stained with alizarin red showed that there is no statistically significant difference among the experimental groups in the number of ossifications in skull, sternum, hyoid, vertebral column (thoracic, lumbar, sacral, and caudal vertebrae), ribs, and bones of the lower limb and upper limbs (Figure 5).

## 4. Discussion

Plant-based traditional medicine has been an alternative option of health care maintenance for so long although the main concern remains safety [28]. There is an obvious misconception by individuals who consumed herbal medicine as all plant-based medications are safe since they are from natural sources. However, recent reports showed that medicinal plants can cause remarkable toxic effects on human wellbeing [29, 30]. Moreover, medicinal plants can cause significant genotoxic, embryotoxic, and teratogenic effects [31, 32]. However, significant number of plants claimed to have medicinal values that are not yet studied for their teratogenic effect. Given this, the current study explored the effect of *E. schimperi* on the developmental indices of rat embryos and fetuses. To the best of our knowledge, there is no published article regarding the teratogenic effect of *E. schimperi* on rat developmental indices. Hence, the current study complements previous efforts to compile the toxicity profile of the plant.

The current study demonstrated that 80% ethanol fruit extract of *E. schimperi* did not exhibit visible clinical symptoms during cage side evaluation of the experimental animals throughout the study period. In agreement with the current studies, Debebe et al. [7] and Zewdu et al. [10] reported that rodents treated with high dose of crude extract of *E. schimperi* showed no behavioral and clinical changes. This might indicate that crude extract of *E. schimperi* is not toxic enough to bring visible clinical manifestations.

Embryonic developmental indices are important indicators to assess the teratogenic effect of different substances [16, 18]. In this regard, the present study affirmed that the crude extract of *E. schimperi* did not affect CRL, number of somites, and morphological score of 12 days old rat embryos. Additionally, there was no difference in the development of circulatory, nervous, and musculoskeletal embryonic systems on both the treatment and control groups. Since the aforementioned developmental indices are directly related to the growth and development of embryos [13], the result of the current study can be suggestive that the crude extract of the plant did not interfere in the process of embryogenesis.

Fetal developmental indices are also relevant touchstones that can describe the extent of developmental delays caused by different agents. These indices were measured in near term rat fetuses (at day-20 of gestation). The present study revealed that there is no dose-related effect of *E. schimperi* fruit on maternal food intake and weight gain throughout the pregnancy period. Concordantly, Zewdu et al. reported that chronic treatment with 80% ethanolic fruit extract of *E. schimperi* shows no significant effect on weight gain. This might be due to a higher safety level of the plant not to interfere and affect food intake as well as weight gain of experimental animals.

According to the current study, the exposure of *E. schimperi* during the active embryogenesis period of pregnant albino Wistar rats did not affect the number of implantations nor did it exhibit prior resorption sites along the length of the gravid uterus. All fetuses were alive at the gestational age of 20 days, the day of sacrifice. These findings might suggest that the crude extract of *E. schimperi* might not affect the implantation process and fetal viability. Furthermore, the current experiment was conducted on young and virgin albino Wistar rats which could be a possible reason for the absence of resorption since the risk of having resorption in rats is higher with old maternal age and elevated body weight [33].

Regarding fetal outcome parameters, the current study disclosed that fetal weight, placental weight, and fetal crown-ramp length were not affected by exposure of the pregnant rats to different doses of the crude extract of *E. schimperi*. This might indicate a higher safety level of the plant extract to influence the aforementioned fetal outcome indices. However, further experiments shall be conducted to rule out possible compensational changes during late gestational periods [13, 19].

The placenta is an important fetomaternal organ that helps the developing fetus to grow properly and safely by being a barrier against some toxic chemicals in addition to its nutritional role. As a result, the placenta becomes a highly susceptible target organ for drug or chemical-induced adverse effects during pregnancy [23]. In this regard, the present study revealed that histopathological analysis of placental tissues from rats treated with 250 mg/kg and 500 mg/kg of *E. schimperi* fruit extract exhibited significant dose dependent inflammatory indices, focal fibropurulent exudate, and hemorrhage when compared to the pair-fed and the ad libitum groups. This might be attributed to possible presence of metabolites like alkaloids and terpenoids causing dose-dependent inflammatory reactions through coagulation cascading and deposition of fibrin in the placental tissue that could provoke impairment of the microvasculature of the placenta [34, 35]. Furthermore, the different scores for the deferent groups might be explained due to the fact that the inflammatory effect of the plant extract is directly proportional to the concentration of the extract. Placental tissue calcification was observed on tissues taken from animals treated with 1000 mg/kg of the fruit extract. One of the possible reasons for placental tissue calcification might be the presence of alkaloids and terpenoids that could provoke the excessive expression of bone morphogenetic protein-7 (BMP7), a transforming growth factor-*β* (TGF-*β*) also known as osteogenic protein-1, near to the implantation site, in the decidua [36]. Another possible hypothesis is the presence of dystrophic calcification, a physiological mechanism by which extracellular calcium combines with phosphate resulting in the formation of hydroxyapatite crystals during apoptosis and tissue perforation caused by trophoblast invasion into phagocyte epithelial and decidual cells [37, 38].

Gross external morphology, visceral morphology, and skeletal evaluations are the key fetal developmental endpoints that were investigated to determine the effect of *E. schimperi* [16, 19]. The present study revealed that there were no significant dose-related differences in gross and visceral fetal morphological indices among *E. schimperi* treatment groups and the control groups. A similar report by Zewdu et al. revealed that chronic treatment with 80% hydroalcoholic extract of *E. schimperi* did not exhibit any sign of toxicity [10]. This indicates that the crude extract of the plant might not bring unintended birth outcomes including overt birth defects.

Another parameter to assess the teratogenic effect of substances such as herbal products in fetal rats is the number of prenatal ossification centers in both axial and appendicular skeletons [16, 39–41]. The current study analyzed the mean number of ossification centers in the sternum, thoracic vertebrae, lumbar vertebrae, caudal vertebrae, ribs, forelimb phalanges, hindlimb phalanges, metacarpus, and metatarsus. However, there was no significant difference in the mean of ossification centers among the treatment groups and their control counterparts. Furthermore, all fetuses exhibited adequate number of ossification centers for their age. This might indicate that the 80% hydroalcoholic fruit extract of *E. schimperi* did not affect osteogenesis of the rat skeleton.

## 5. Conclusion

The results of this study revealed that the administration of 80% hydroalcoholic extract of *E. schimperi* fruit during the period of organogenesis in rats did not exhibit a significant teratogenic effect on embryonic and fetal developmental indices. However, histopathological examination of the placenta showed inflammatory reactions and calcifications on the maternal part of the rat placenta. This is a redolent scenario that the extract affects the microvasculature of the placenta. Therefore, the consumption of *E. schimperi* fruits by pregnant women is not recommended before ensuring the safety of the plant for human consumption especially without further investigation of inflammatory mechanism in the placenta. Hence, the authors would like to recommend further *in-vivo* studies to investigate the histopathological perspectives of placentas from *E. schimperi* treated animals. We also strongly recommend to conduct further safety studies of the plant in rodents other than rats.

## Figures and Tables

**Table 1:** Embryonic outcome variables across experimental groups after treatment with the 80% ethanol fruit extract of *Embelia schimperi*.

Group	*Embryonic developmental outcomes*		
	Morphological score/litter	Number of somites/litters	CRL (mm)/litter
Group I (250 mg/kg)	44.71 ± 0.7	27.91 ± 0.4	4.6 ± 0.3
Group II (500 mg/kg)	44.52 ± 0.8	27.41 ± 0.3	4.4 ± 0.3
Group III (1000 mg/kg)	44.32 ± 0.8	26.99 ± 0.4	4.5 ± 0.2
Group IV (pair-fed)	44.44 ± 0.6	27.23 ± 0.5	4.4 ± 0.4
Group V (ad libitum)	44.63 ± 0.7	27.43 ± 0.4	4.6 ± 0.4

Results are expressed in terms of the mean and the standard deviation from the mean; one way ANOVA, CRL: crown-ramp length.

**Table 2:** Embryonic development developmental indices of the circulatory system after treated with 80% ethanol fruit extract of *Embelia schimperi*.

Group	Proportion of retarded development		
	Yolk sac circulation	Heart	Allantois
Group I (250 mg/kg)	0	0	0
Group II (500 mg/kg)	0	0	0
Group III (1000 mg/kg)	0	0	0
Group IV (pair-fed)	0	0	0
Group V (ad libitum)	0	0	0

Results are expressed in terms of proportions retarded development (%), chi-square test.

**Table 3:** Embryonic developmental indices of the nervous system and sense organs after treatment with the 80% ethanol fruit extract of *Embelia schimperi*.

Group	Proportion of retarded development				
	Caudal neural tube	Hind brain	Fore brain	Otic system	Optic system
Group I (250 mg/kg)	0	0	0	0	0
Group II (500 mg/kg)	0	0	0	0	0
Group III (1000 mg/kg)	0	0	0	0	0
Group IV (pair-fed)	0	0	0	0	0
Group V (ad libitum)	0	0	0	0	0

Results are expressed in terms of proportion retarded development (%), chi-square test.

**Table 4:** Embryonic developmental indices of the musculoskeletal system after treatment with the 80% ethanol fruit extract of *Embelia schimperi*.

Group	*Proportion of retarded development*					Flexion
	Pharyngeal apparatus	Maxillary process	Mandibular process	Fore limb	Hind limb	
Group I (250 mg/kg)	0	0	0	0	0	0
Group II (500 mg/kg)	0	0	0	0	0	0
Group III (1000 mg/kg)	0	0	0	0	0	0
Group IV (pair-fed)	0	0	0	0	0	0
Group V (ad libitum)	0	0	0	0	0	0

Results are expressed in terms of proportions retarded development (%), chi-square test.

**Table 5:** Food intake and weight gain of pregnant rats treated with 80% ethanol fruit extract of *Embelia schimperi*.

Maternal variables	*Experimental groups*				
	Group I (250 mg/kg)	Group II (500 mg/kg)	Group III (1000 mg/kg)	Group IV (pair-fed control)	Group V (ad libitum)
Food intake (g)	172.94 ± 35.26	183.09 ± 22.36	184.74 ± 8.63	180.26 ± 18.08	174.57 ± 33.92
Weight gain (g)	77.55 ± 3.27	85.73 ± 6.97	91.91 ± 5.91	81.1 ± 7.31	88.6 ± 8.21

NB: Results are expressed as the mean ± standard deviation from the mean, one-way ANOVA.

**Table 6:** Pregnancy outcome of rats treated with 80% ethanol fruit extract of *Embelia schimperi*.

Pregnancy outcomes	*Experimental groups*				
	Group I (250 mg/kg)	Group II (500 mg/kg)	Group III (1000 mg/kg)	Group IV (pair-fed control)	Group V (ad libitum)
Number of implantation/dams	10 ± 2.16	11.5 ± 1.73	10.8 ± 1.52	9.5 ± 1.29	9 ± 1.63
Number of prior resorptions/dams	0	0	0	0	0
Alive pups	10 ± 2.16	11.5 ± 1.73	10.8 ± 1.52	9.5 ± 1.29	9 ± 1.63
Dead pups	0	0	0	0	0

NB: Results are expressed as the mean ± standard deviation from the mean, one-way ANOVA.

**Table 7:** Fetal outcome of rats treated with 80% ethanol fruit extract of *Embelia schimperi*.

Fetal outcomes	*Experimental groups*				
	Group I (250 mg/kg)	Group II (500 mg/kg)	Group III (1000 mg/kg)	Group IV (Pair-fed control)	Group V (ad libitum)
Fetal weight (g) per dam	3.68 ± 1.04	3.94 ± 0.99	3.98 ± 0.82	3.97 ± 1.01	3.91 ± 0.99
Placental weight (g)	0.61 ± 0.08	0.62 ± 0.04	0.54 ± 0.19	0.61 ± 0.06	0.59 ± 0.07
Crown-ramp length (cm)	4.75 ± 0.26	4.80 ± 0.39	3.67 ± 0.18	4.53 ± 0.85	4.44 ± 0.59

NB: Results are expressed as the mean ± standard deviation from the mean, one-way ANOVA.

**Table 8:** Distribution of placental histopathological manifestations across experimental groups.

Histopathological parameters of placenta	*Experimental groups*				
	Group I (250 mg/kg)	Group II (500 mg/kg)	Group III (1000 mg/kg)	Group IV (pair-fed control)	Group V (ad libitum)
Necrosis	0	0	0	0	0
Cytolysis	0	0	0	0	0
Apoptosis	0	0	0	0	0
Inflammation	30^*∗*^	40^*∗*^	60^*∗*^	0	0
Calcification	0	0	30^*∗*^	0	0

NB: Results are presented as percentages of histopathological findings; ^*∗*^statistically significant difference seen from the ad libitum and control at *P* < 0.05, chi-square test.

**Table 9:** External gross malformations after treatment with the 80% ethanol fruit extract of *Embelia schimperi*.

Group	*Proportion of external malformations (%)*							
	*Nervous system defects*			*Musculoskeletal defects*			*Others*	
	ExE	AnE	SB	KY	SC	LD	MT	EGA
Group I (250 mg/kg)	0	0	0	0	0	0	0	0
Group II (500 mg/kg)	0	0	0	0	0	0	0	0
Group III (1000 mg/kg)	0	0	0	0	0	0	0	0
Group IV (pair-fed)	0	0	0	0	0	0	0	0
Group V (ad libitum)	0	0	0	0	0	0	0	0

Results are expressed in terms of proportions of malformations, chi-square test; ExE: exencephaly, AnE: anencephaly, SB: spina bifida, KY: kyphosis, SC: scoliosis, LD: limb defect, MT: missed tail, and EGA: external genitalia agenesis.

**Table 10:** Number of ossification centers in the axial skeleton of rat fetuses from experimental groups treated with the 80% ethanol fruit extract of *Embelia schimperi*.

Group	Sternum	Thoracic vertebrae	Lumbar vertebrae	Caudal	Ribs
Group I (250 mg/kg)	5.61 ± 0.32	12 ± 0	5 ± 0	4.38 ± 0.96	24 ± 0
Group II (500 mg/kg)	5.60 ± 0.27	12 ± 0	5 ± 0	3.94 ± 0.99	24 ± 0
Group III (1000 mg/kg)	5.62 ± 0.30	12 ± 0	5 ± 0	3.81 ± 1.17	24 ± 0
Group IV (pair-fed)	5.63 ± 0.28	12 ± 0	5 ± 0	4.38 ± 1.20	24 ± 0
Group V (ad libitum)	5.62 ± 0.33	12 ± 0	5 ± 0	4.31 ± 1.01	24 ± 0

Results are presented as the mean ± standard deviation from the mean of ossification centers count, one-way ANOVA.

**Table 11:** Number of ossification centers in the appendicular skeleton of rat fetuses from experimental groups treated with the 80% ethanol fruit extract of *Embelia schimperi*.

Group	Forelimb phalanges	Hindlimb phalanges	Metacarpus	Metatarsus
Group I (250 mg/kg)	3.77 ± 0.32	3.51 ± 0.42	3.9 ± 0.31	4.09 ± 0.31
Group II (500 mg/kg)	3.83 ± 0.26	3.47 ± 0.37	3.8 ± 0.41	4.02 ± 0.22
Group III (1000 mg/kg)	3.67 ± 0.43	3.43 ± 0.51	3.9 ± 0.26	3.99 ± 0.33
Group IV (pair-fed)	3.91 ± 0.28	3.61 ± 0.33	3.93 ± 0.24	4.13 ± 0.18
Group V (ad libitum)	3.88 ± 0.41	3.73 ± 0.29	3.87 ± 0.35	4.17 ± 0.02

Results are presented as the mean ± standard deviation from the mean of ossification centers count, one-way ANOVA.

## Data Availability

All the data utilized in this study are found in the manuscript.

## Abbreviations

ANOVA: Analysis of variance
CRL: Crown-ramp length
EPHI: Ethiopian Public Health Institute
IRB: Institutional Review Board
LD50: Mean lethal dose
OECD: Organization for Economic Co-operation and Development
SD: Standard deviation
SPSS: Statistical Package for the Social Sciences
TMMRD: Traditional and Modern Medicine Research Directorate

## Supplementary Materials

Supplementary Materials Supplemental file: Brown and Fabro morphological scoring parameters are attached as a supplemental file.
