# Supplementary material for: Teratogenic Evaluation of 80% Ethanol Extract of Embelia schimperi Vatke Fruits on Rat Embryo and Fetuses
Source: J Toxicol. 2022 Oct 22;2022:4310521. doi: 10.1155/2022/4310521 (PMC9617728; doi:10.1155/2022/4310521)
Supplement: Supplementary Materials — Supplemental file: Brown and Fabro morphological scoring parameters are attached as a supplemental file. [file 4310521.f1.docx]

Brown and Fabro morphological scoring system for rat embryos

|  | 0 | 1 | 2 | 3 | 4 | 5 | Score |
| --- | --- | --- | --- | --- | --- | --- | --- |
| Yolk sac circulation system | not visible or scattered island | corona of blood islands with/without anastomosis | vitelline vessel with few yolk sac vessels | full yolk sac plexus of vessels | yolk sac obliterated vitelline artery and vein well separated |  |  |
| Allantois | allantois free in exoceolom | allantois fused with chorion | umbilical vessels | separate aortic origin of umbilical and vit vessels |  |  |  |
| Flexion | ventrally convex | Turning | dorsally convex | dorsally convex with spiral torsion |  |  |  |
| Heart | endocardial rudiment not visible or visible but not beating | beating "s "shaper cardiac tube | convoluted cardiac tube | bulbus cordis,atrium commune or ventriculur communes | dividing atrium communes |  |  |
| Caudal neural tube | Neural plate or fold | closing but unfused neural fold/groove | neural fold closed at level of somite4/5 | posterior neuropore formed but open | posterior neuropore closed |  |  |
| Hind brain | Neural plate | rhombomere A and B | anterior neuropore formed but open | anterior neuropore closed rhomboncephalon formed | pronounced pontine flexure with transparent roof of 4th ventricle |  |  |
| Midbrain | Neural plate | mesencephalic brain folds | closing of mesencephalic folds | completely fused mesencephalon | visible division b/n mesencephalon and diencephalon |  |  |
| Forebrain | Neural plate | prosencephalic brain folds | completely fused prosencephalon | visible telencephalic evagination | well elevated telencephalic hemisphere |  |  |
| Otic system | no sign of otic dev’t | flattened otic primordium | otic pit | otocyst | otocyst with dorsa recess | otocyst with endo lymphatic duct |  |
| Optic system | no sign of optic dev’t | sulcus opticus | elongated optic primordium | primary optic vesicle with open optic stalk | indented lens plate | lens pocket or vesicle |  |
| Olfactory system | No sign of olfactory dev’t | olfactory plate | olfactory plate with rim | distinct olfactory ridge | lateral nasal process and medial rim |  |  |
| Branchial bars | none visible | I visible | I and II visible | I, II and II visible | II overgrowing and obscure III |  |  |
| Maxillary process | No sign of maxillary dev’t | Maxillary process demarcated. Visible cleft anterior to bar I | Maxillary process fused with nasal process |  |  |  |  |
| Mandibular process | No sign of mandibular dev’t from bar I | First branchial bar fused and forming mandibular process |  |  |  |  |  |
| Fore limb | No sign of fore limb dev’t | Distinct evagination of wolfian crest at the level of somite 9-13 | Forelimb bud | Paddle shaped for limb bud | Distinct apical ridge on forelimb |  |  |
| Hind limb | No sign of hind limb dev’t | Distinct evagination of Wolfian crest  at level 01 somite’s 26-30 | Hind limb bud | Paddle shaped for hind bud |  |  |  |
| Somite’s | 0-6 | 7-13 | 14-20 | 22-27 | 28-34 | 35-41 |  |
